# SYNCHRONOUS ANAL MELANOMA AND COLON ADENOCARCINOMA: CASE REPORT AND REVIEW OF DIAGNOSIS AND MANAGEMENT

**DOI:** 10.1590/0102-6720201600040020

**Published:** 2016

**Authors:** Eduardo Henrique PIROLLA, Felipe Piccarone Gonçalves RIBEIRO, Fernanda Junqueira Cesar PIROLA, Camila COSMO, Melany Di BIASI

**Affiliations:** 1 Harvard Medical School, Boston, Massachusetts, USA;; 2 Postgraduate Program in Interactive Process of Organs and Systems, Federal University of Bahia, Salvador, Bahia, Brazil;; 3 Center for Technological Innovation in Rehabilitation, Federal University of Bahia, Salvador, Bahia, Brazil

**Keywords:** Melanoma, Adenocarcinoma, Diagnosis

## INTRODUCTION

Malignant anal melanoma is a rare disorder, corresponding to 0.05-1.0% of all anorectal tumors, and 0.4-1.6% of all other melanomas^7^ ^,^ ^8^ ^,^ ^9^. Its rarity can be confirmed by the fact that for every anal melanoma, there are eight squamous cell carcinomas and 250 anal adenocarcinomas^8^ ^,^ ^9^.

The article´s aim is to present a case of a malignant anal melanoma coexisting with colon adenocarcinoma, in addition to a discussion on how to speed up the diagnosis with simple routine measures, and report an objective treatment.

## CASE REPORT

A 57-year-old patient was admitted with weakness, pale skin and a lump in the inguinal region. According to his medical history, one year prior to admittance the patient was treated for anemia. At the time, he presented positive fecal occult blood test. Endoscopy and contrast exams were normal and no weight loss or changes in bowel movements were noticed. 

In the six months prior to admittance, the patient felt sporadic pain in the anal canal that ceased with the use of NSAID suppositories. After 30 days, he sought medical attendance and underwent proctosigmoidoscopy and colonoscopy, along with biopsy of lesions in the anal canal and cecum. The patient was then diagnosed with poorly differentiated carcinoma of the canal and well differentiated tubular adenocarcinoma of the cecum. At the time, a lump began forming in the root of the thigh, just below the inguinal fold. 

**FIGURE 1:**
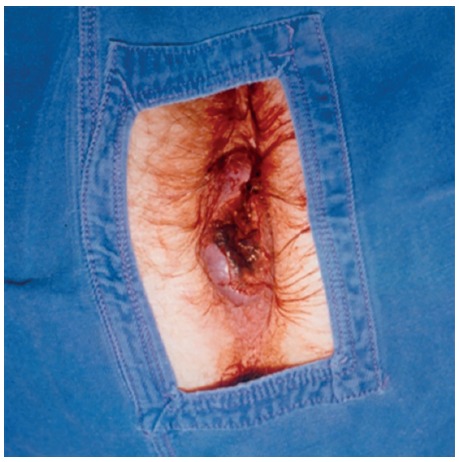
Circunferencial anal lesion in all anorectal canal

The patient was refered to Sírio-Libanês Hospital in São Paulo, Brazil, for surgical treatment of the colon lesion and clinical treatment of the anal canal lesion (Figure 1), as he refused to operate the anal lesion. Abdominal ultrasonography and thorax tomography did not reveal any findings. Surgery was then performed with right ileocecal colectomy, intra-operatory biopsy of lesion in the anal canal and of the right inguinal lymph node. 

**FIGURE 2:**
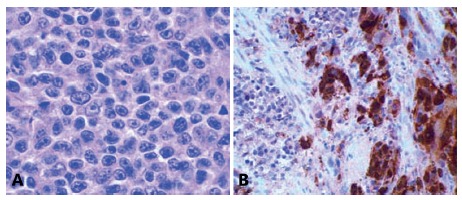
A) Amelanotic epithelioid type (H&E) and B) epithelioid/lymphoma-like (Study S-100 protein)

Anatomopathological findings of the anal canal revealed an amelanotic malignant melanoma with extensive infiltration and presence of ulcerations and necrosis (Figure 2); right inguinal lesion was a metastatic amelanotic malignant melanoma.

Findings on the colon lesion revealed a segment of the left colon had histological stage II invasive tubular adenocarcinoma, a presence of 20% of mucous membrane, diffuse and extensive infiltration of the colon wall up to the peripheral adipose tissue, ulcerous lesion with irregular areas and necrosis, discreet fibrosis and inflammatory infiltration, and absence of vascular or peri-neural invasion. The anatomopathological examination of surgical borders revealed absence of neoplasm. Assessment of mesocolic lymph nodes exhibited reactive lymphoid hyperplasia with no metastasis (0/13). 

The patient refused surgical treatment on the anal canal and neglected follow-up. After two months, he returned with exophytic lesion and bleeding, along with significant loss of weight and pain. In the 30 days before hospitalization he presented important anal bleeding with spontaneous remission. In the 6^th^ day prior to hospitalization, there was a new episode of bleeding, which led to hospitalization. A new abdominal tomography showed multiple adenomegalies in the paraaortic, paracaval, inguinal, and bilateral perirectal lymph nodes. It also revealed a heterogenic mass with imprecise limits, compatible with ganglion conglomerates of mesenteric origin, next to the hepatic angle of the colon; presence of a soft tissue mass in the anal canal, extending to the ischiorectal fossa towards the perineal border, predominantly on the left, with no dividing plane with the elevator muscle of the anus. Due to the bleeding and ulceration, a colonostomy was performed and local radiotherapy began. After the third session of 300 rads in each there was significant improvement of the bleeding, which stopped after the fourth session. There was clinical improvement and the patient is currently undergoing monitoring.

## DISCUSSION

Malignant anorectal melanoma was first described by Moore in 1857^1^. The origin of the tumor appears to be ectodermic, although the presence of melanocytes in the colorectal, above the squamous and transitional regions of the anal canal, proves that the tumor can originate above just as bellow the pectinate line^1^ ^,^ ^2^ ^,^ ^11^. Some authors argue that, in almost all cases, the tumor originates in the pectinate line and grows in the submucous space, scrolling through the tissue to emerge on the mucosa at a higher point, thus simulating a primary rectal tumor^2^ ^,^ ^6^.

The prognosis is poor since the survival rate is low^5^ ^,^ ^8^ ^,^ ^13^. Some authors relate this to a late diagnosis in most cases^1^ ^,^ ^3^ ^,^ ^12^.

The early signs and symptoms are similar to other colorectal diseases, being most frequent anal bleeding, local pain, changes in bowel habits, and in many times, the growth of lumps in the region^1^ ^,^ ^3^ ^,^ ^8^ ^,^ ^12^. The difficulty in diagnosing is brought by the similarities of the symptoms with hemorrhoidal disease or anal fissure, as well as the fact that in 16-41% of lesions lack pigmentation^1^ ^,^ ^8^ ^,^ ^12^.

The incidence is greater in women^1^ ^,^ ^8^ ^,^ ^12^. The average age group consists of patients in their sixties and seventies^5^ ^,^ ^6^ ^,^ ^12^. 

Resection is the treatment of choice among the authors depending on the size of the lesion. The level of infiltration and presence of metastases are some of the factors considered when choosing the extension of resection^1^ ^,^ ^9^. Previous or adjuvant postoperative radiotherapy and chemotherapy is very debatable.

Many authors believe that the origin of these tumors is in the dentate line, where melanocytes have been detected^1^ ^,^ ^2^ ^,^ ^8^. Iron ions have been used for staining, currently; the Masson-Fontana technique is used to show melanocytes above the dentate line^2^ ^,^ ^5^ ^,^ ^8^. The most up-to-date techniques use immunohystochemical reactions such as S-100 protein (antibodies S-100), which has greater sensibility, but less specificity. Monoclonal antibodies are also used as a detection method, such as HMB-45 (monoclonal Enzo)^2^.

The Breslow classification is best used to classify the depth of the tumor^1^ ^,^ ^8^. This method is preferred to the Clark classification, regarding the lack of papillary dermis in the region^1^ ^,^ ^8^.

Many authors classify anorectal melanomas in stages; the most common being: stage I - in situ; stage II - regional spread (inguinal lymphadenopathy); stage III - metastasis^1^ ^,^ ^3^.

The metastasis areas can be arranged according to its frequency: lungs, bones, liver, brain and gastrointestinal tract^1^ ^,^ ^3^.

The early diagnosis is essential to improve the prognosis of such an aggressive disease. Survival rates vary from 6-12% for five years^4^ ^,^ ^7^ ^,^ ^10^. The average survival rate in literature is 18 months^12^.

Some factors can worsen the prognosis such as late diagnosis, ulceration, rich mucous vascularization, and aggressive nature of the tumors. 

Since the most common complaints are enterorrhagia and anal bleeding, it is essential to perform a proctosigmoidoscopy and biopsy^7^ ^,^ ^8^ ^,^ ^9^. The pathologist should be reminded of the hypotheses, for there might be difficulties in differential diagnosis of anaplastic carcinoma and hemorrhoidal disease. It is essential to obtain other anatomopathological evidence. Some authors recommend using contrast exams along with colonoscopy^3^ ^,^ ^7^ ^,^ ^12^.

The malignant anorectal melanoma presents early metastases^1^. Mesenteric lymph nodes involvement is more common than inguinal^1^. Rectum lesions drain to the mesorectal lymph nodes and to the inferior mesenteric chain, which can occur in 33.3% of the cases. Cutaneous anal lesions drain to the superficial inguinal lymph nodes^1^. The dissemination through hematogenous pathway can reach the liver, lungs, bones, brain and gastrointestinal tract^1^. Authors diverge regarding conduct and treatment, although the recommended procedure is surgery, there are debates regarding local excision or abdominoperineal resection, and another group of authors that indicates radiotherapy and/or chemotherapy to control signs and symptoms, such as bleeding, regarding intracavitary and inguinal lymph nodes. 

For some authors^1^ ^,^ ^6^ abdominoperineal resection can lead to a greater survival rate, especially if there is no lymph node invasion (Stage I). 

The great majority of authors did not show significant difference in survival rate in patients referred to abdominoperineal resection and local excision ^1^ ^,^ ^3^. There is some consensus on the fact that less recurrence is observed when abdominoperineal resection is performed, but with no statistical significant^3^.

Another aspect is that patients without compromised lymph nodes who are refered to abdominoperineal resection have a more favorable prognosis when compared to cases with compromised lymph nodes^6^. Some authors believe that the prognosis is strictly related to the aggressive nature and early surgery^4^.

Regarding adjuvant treatments, literature contests the success of radiotherapy (anorectal muscle non-responsiveness), as well as the lack of clarity of chemotherapy success^4^. Radiotherapy and hyperthermia is mentioned to control in situ disease, followed by a regression of the tumor^12^. Radiotherapy is not the treatment of choice, but it can be used as palliative treatment^12^. Immunotherapy is another method, being administered to patients that become surgically free (macroscopically) of the disease. This treatment is carried out for 4-7 months with subcutaneous injections of irradiated melanocytes with Calmette-guerin bacilli.

In conclusion, studies show the highly aggressive nature of the tumor, and the difficulty in early diagnosis and initial clinical staging. The prognosis is poor and patients face challenging metastases^3^. Surgical outcomes are mediocre and very few live over five years^1^.
